# Strong CD8+ lymphocyte infiltration in combination with expression of HLA class I is associated with better tumor control in breast cancer patients treated with neoadjuvant chemotherapy

**DOI:** 10.1007/s10549-019-05195-y

**Published:** 2019-03-13

**Authors:** A. F. de Groot, E. J. Blok, A. Charehbili, C. C. Engels, V. T. H. B. M. Smit, N. G. Dekker-Ensink, H. Putter, E. Meershoek-Klein Kranenbarg, C. J. H. van de Velde, G. J. Liefers, J. W. R. Nortier, P. J. K. Kuppen, S. H. van der Burg, J. R. Kroep

**Affiliations:** 1 Department of Medical Oncology, Leiden University Medical Center, Albinusdreef 2, 2333 ZA Leiden, The Netherlands; 2 Department of Surgery, Leiden University Medical Center, Albinusdreef 2, 2333 ZA Leiden, The Netherlands; 3 Department of Pathology, Leiden University Medical Center, Albinusdreef 2, 2333 ZA Leiden, The Netherlands; 4 Department of Statistics, Leiden University Medical Center, Albinusdreef 2, 2333 ZA Leiden, The Netherlands

**Keywords:** Neoadjuvant chemotherapy, Breast cancer, Zoledronic acid, Tumor-infiltrating lymphocytes, HLA class 1, Pathological complete response

## Abstract

**Purpose:**

Tumor-infiltrating lymphocytes (TILs) are associated with pathological complete response (pCR) and survival after neoadjuvant chemotherapy (NAC) in patients with early breast cancer. We investigated the prognostic and predictive role of TILs, macrophages, and HLA class 1 expression after NAC with or without the potentially immune modulating compound zoledronic acid (ZA).

**Methods:**

Baseline tumor biopsies from 196 patients in the NEOZOTAC trial were analyzed for CD8 (cytotoxic T-cells), FoxP3 (regulatory T-cells), CD68 (macrophages), and HLA class I (HCA2/HC10) expression by immunohistochemistry and subsequently related to pCR and disease-free survival (DFS).

**Results:**

A strong intratumoral CD8+ infiltration or expression of HLA class 1 by cancer cells was associated with a higher pCR rate (*p* < 0.05). Clinical benefit of high CD8+ T-cell infiltration was found when cancer cells expressed HLA class 1 (pCR: 21.8% vs. 6.7%, *p* = 0.04) but not when HLA class 1 expression was lost or downregulated (pCR: 5.9% vs. 0%, *p* = 0.38). Interaction analyses revealed survival benefit between HLA class 1 expression and strong CD8+ T-cell infiltration, whereas in the absence or downregulation of HLA class 1 expression, high levels of CD8+ T-cells were associated with survival disadvantage (p for interaction 0.01; hazard ratio 0.41, 95% CI 0.15–1.10, *p* = 0.08 and hazard ratio 7.67, 95% CI 0.88–66.4, *p* = 0.07, respectively). Baseline immune markers were not related to ZA treatment.

**Conclusions:**

Strong baseline tumor infiltration with CD8+ T-cells in the presence of tumoral HLA class 1 expression in patients with HER2-negative breast cancer is related to a higher pCR rate and a better DFS after NAC.

**Electronic supplementary material:**

The online version of this article (10.1007/s10549-019-05195-y) contains supplementary material, which is available to authorized users.

## Background

For patients with locally advanced breast cancers, neoadjuvant chemotherapy (NAC) is an increasingly used treatment modality. The main goal of neoadjuvant therapy is to enable breast-conserving therapy and spare patients from an axillary lymph node dissection. Additionally, neoadjuvant studies make use of the possible accelerated drug selection for evaluation in adjuvant phase III early breast cancer trials [1]. Studies have shown that the occurrence of pathological complete response (pCR) after neoadjuvant therapy is associated with improved survival, especially in non-luminal type tumors [2–4].

Evaluation of tumor-infiltrating lymphocytes (TILs) on both hematoxylin and eosin (H&E) stained and immunohistochemical stained tumor sections has demonstrated promising value as a predictor of pCR and survival after neoadjuvant therapy, especially in patients with estrogen receptor (ER)-negative and HER2-positive disease [5–7], and efforts are being made for harmonization of this parameter by a dedicated international TIL-working group [8–10]. However, the composition of TILs is typically heterogeneous and includes cytotoxic T-cells (CTLs), targeting tumor cells, as well as immunosuppressing regulatory T-cells (Tregs) with potentially different implications for the chances of achieving pCR and improving survival [11, 12]. A recent meta-analysis by Wang et al. confirmed the findings of several studies by showing a positive association between higher levels of CD8+ CTL infiltrate and pCR [5]. Data on the relation between CD8+ CTL infiltrate and survival after NAC are less extensive, but some studies suggest high CD8+ CTL infiltrate to be prognostic for an improved survival [13–15]. For FoxP3+ Treg infiltrate, meta-analyses by Wang et al. and Mao et al. reported contradictory results regarding an association with pCR [5, 16] and also studies on an association with survival reported discordant results [15, 17–19]. Tumor-associated macrophages (TAMs) may also play a role in the tumor microenvironment [20], but studies on (CD68+) TAM infiltrate as a biomarker in the neoadjuvant setting are scarce [21, 22]. In general, it is thought that patients with high numbers of TAM infiltrate are likely to have a worse survival rate [20]. Importantly, tumor cells can acquire features to escape from immune recognition by downregulation of HLA class 1 expression [23]. Previously, de Kruijf et al. observed that loss and downregulation of HLA class 1 resulted in worse outcome in terms of breast cancer relapse [12]. However, only few studies have investigated the role of HLA class 1 expression and results are discordant [12, 24–26].

In this study, we aimed to investigate the role of CTLs, Tregs, and TAMs in the tumor microenvironment in the context of HLA class 1 expression in a study cohort consisting of patients treated with NAC with or without zoledronic acid (ZA) in a randomized phase III trial [27]. As ZA is suggested to have immunomodulatory features [28], our secondary aim was to investigate whether there were differential treatment benefits of ZA between biomarker-based subgroups with regard to pCR and disease-free survival (DFS).

## Methods

### Patients and tumor material

Eligibility criteria for the NEOZOTAC study have been described previously [27]. In brief, patients with stage II/III, HER2-negative breast cancer were randomized to 6 cycles of NAC with or without ZA. Medical-ethical approval was obtained and the study was conducted in accordance with the Declaration of Helsinki. All patients signed informed consent before randomization. Formalin-fixed paraffin-embedded (FFPE) samples were collected from pre-NAC biopsies. All samples were handled in a coded fashion, according to national ethical guidelines (“Code for Proper Secondary Use of Human Tissue,” Dutch Federation of Medical Scientific Societies). Central data collection was done at the Datacenter of the Department of Surgery of the LUMC. Biomarkers were reported according to the REMARK criteria [29].

### Antibodies

Mouse monoclonal antibodies against CD8 (ab17147 clone 144B; Abcam (1:25) and clone 4B11; Monosan (1:25)), FoxP3 (ab20034 clone 236A/E7; Abcam (1:200)), and CD68 (M0814 clone KP1; Dako (1:15.000)) were used for immunohistochemical identification of CTLs, Tregs, and macrophages, respectively. For HLA class 1, mouse monoclonal antibodies HCA2 (1:200) and HC10 (1:400) were used. The HCA2 antibody binds to all HLA-A chains (except HLA-A24) and some of the HLA-B/HLA-C/HLA-E/HLA-F/HLA-G chains [30, 31]. The HC10 antibody binds to HLA-B, HLA-C, and some HLA-A chains (HLA-A3/HLA-A10/HLA-A28/HLA-A29/HLA-A30/HLA-A31/HLA-A32/HLA-A33) [32–34]. Antibodies were kindly provided by Prof. Dr. J. Neefjes.

### Immunohistochemistry

Immunohistochemical stains were performed on 4-µm sections from FFPE blocks with core needle tumor biopsies. Tissue sections were deparaffinized and rehydrated. Slides were peroxidase blocked for 20 min in H_2_0_2_ solution (0.03%). Antigen retrieval was performed in PT link (Dako) (low pH for CD8/CD68, high pH for FoxP3) for 10 min. After incubation with secondary antibody envision anti-mouse (Dako Cytomation K4001), sections were visualized using 3,3′-diaminobenzidine (DAB+; K3468, Dako). Sections were counterstained with hematoxylin, dehydrated and mounted in Pertex. For HCA2/HC10, stains were performed using the DAKO autostainer. Human tonsil or placenta tissue served as a positive control for each staining. Slides that underwent the complete immunohistochemical staining procedure without primary antibody served as negative controls.

### Evaluation of immunostaining

Microscopic analyses of HCA2, HC10, and FoxP3+ lymphocyte counts were performed blinded by two independent observers (A.C. 100% of cohort, C.E. 30% of cohort). Analyses of CD8+ lymphocyte and CD68+ macrophage counts were performed using the Vectra 3.0, an automated quantitative pathology imaging system with InForm software (PerkinElmer, Waltham MA).

The percentage of tumor cells showing membranous staining for HCA2/HC10 was estimated. Expression status of HLA class 1 was defined according to the International HLA and Immunogenetics Workshop: downregulation of tumor cell HLA class 1 expression was defined as less than 5% of tumor cells staining positive for either HCA2 or HC10 and loss of tumor cell HLA class 1 expression as less than 5% of tumor cells staining positive for both HCA2 and HC10 [35]. A dichotomous variable for loss + downregulation (HCA2 and/or HC10 < 5%) vs. expression (HCA2 and HC10 ≥ 5%) was calculated.

Quantification of the number of tumoral FoxP3+ lymphocytes was done by assessment of three random tumor-containing fields at a × 20 magnification. For quantification, a reticle grid (incorporated in the microscope ocular) with 1 square representing 0.36 mm^2^ was used for manual counting of positive lymphocytes. The lymphocyte counts in 3 squares were averaged for the lymphocyte score. A variable for FoxP3 status (high/low) was calculated using the median as cut-off value.

In brief, for quantification of the number of CD8+ and CD68+ cells, slides were scanned for image acquisition (× 4 magnification). Subsequently, ten multispectral images per slide on average were selected manually (× 20 magnification, one image representing roughly 0.33 mm^2^). With InForm software, the computer was trained to segment stroma, tumor, and other tissue (e.g., adipose tissue/empty spaces) and to distinguish between DAB-positive CD8/CD68 cells and DAB-negative cells with a learn-by-example interface. The resulting algorithm was tested for accuracy in several images before being used for all images, where after the number of CD8+ or CD68+ cells per mm^2^ tumor could be calculated. A variable for CD8 and CD68 status (high/low) was calculated using the median as cut-off value. The creation of the algorithms was done by two researchers (AG/EB) under supervision of a pathologist (VS).

### Statistical analyses

Statistical analyses were performed using SPSS (v.23.0 for Windows, IBM SPSS statistics). Patients of whom tumor material was lost during or appeared non-evaluable after staining were excluded from analyses. Cohen’s kappa coefficient was used to assess inter-observer agreement in manual quantification of immunohistochemical stains (HCA2/HC10/FoxP3). Clinical endpoints examined were pCR, defined as absence of residual tumor in the breast and lymph nodes, and DFS, defined as time from randomization to first relapse, second primary invasive breast cancer or death (any cause). Associations between markers and pCR were tested using Chi-square and Fisher’s exact tests. Survival analyses were performed using Kaplan–Meier curves and Cox regression analyses. Median follow-up time (4.6 years) was determined using the reverse Kaplan–Meier method. For multivariable analyses, logistic regression was used.

## Results

**Fig. 1:**
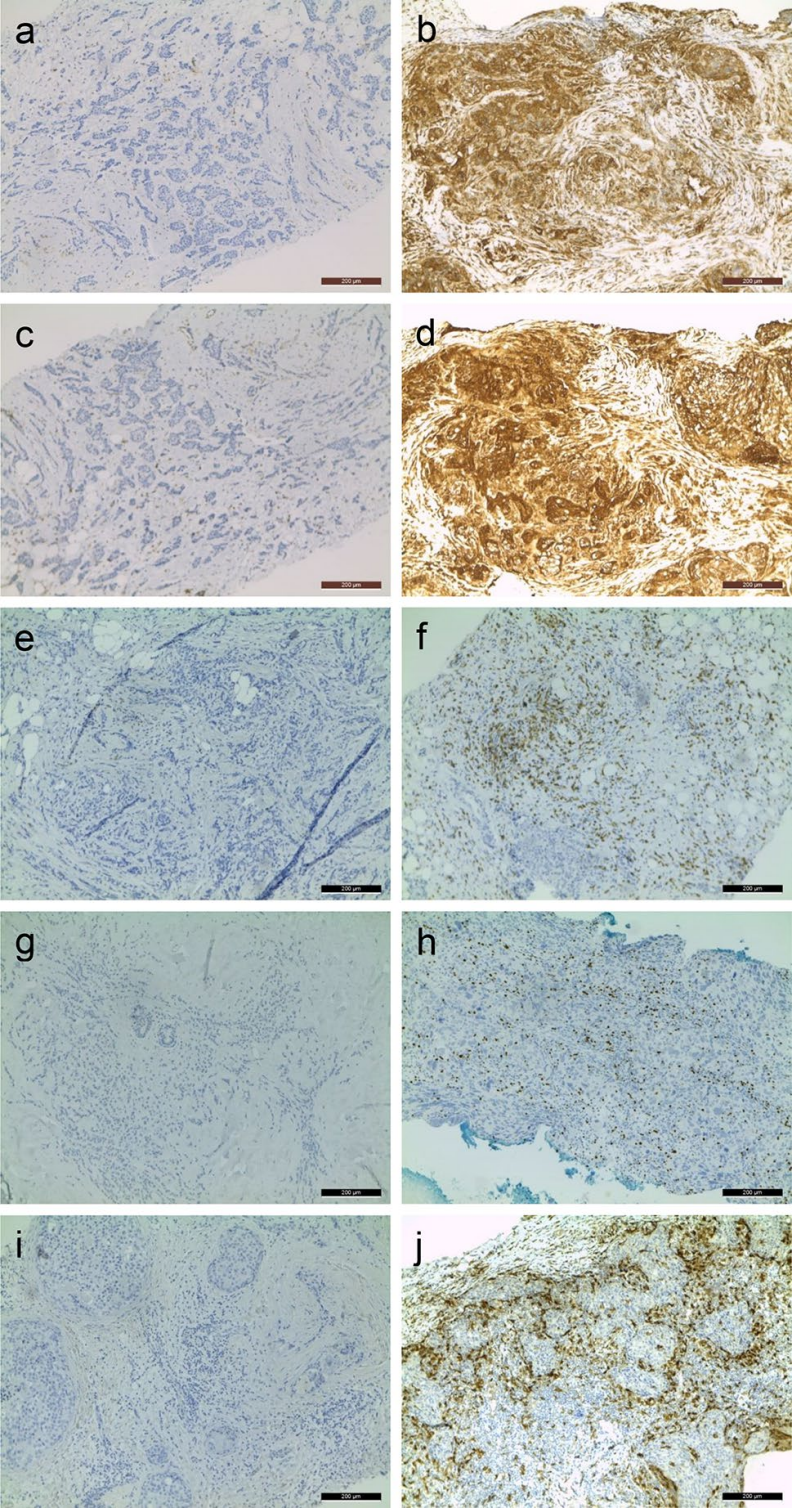
Representative images of immunohistochemical stains for HLA class 1 (HCA2/HC10), CD8, FoxP3, and CD68. **a** HCA2-negative tumor (< 5% of tumor cells staining positive for HCA2). **b** HCA2-positive tumor (≥ 5% of tumor cells staining positive for HCA2). **c** HC10-negative tumor. **d** HC10-positive tumor. **e** Tumor with low (below median) CD8+ infiltrate. **f** Tumor with high (above median) CD8+ infiltrate. **g** Tumor with low FoxP3 infiltrate. **h** Tumor with high FoxP3+ infiltrate. **i** Tumor with low CD68+ infiltrate. **j** Tumor with high CD68+ infiltrate. Images were taken at × 10 magnification

Of 196 out of 250 patients, core biopsy material before treatment was available for evaluation. Patient and tumor characteristics are described in Table 1. An overview of these characteristics for each separate staining can be found in Online Resource 1. Substantial agreement (kappa ≥ 0.6) was observed for quantification of the immunohistochemical stains for FoxP3, HCA2, and HC10. Representative images of immunohistochemical stains for HLA class 1, CD8, FoxP3, and CD68 are shown in Fig. 1.

**Table 1:** Patient and tumor characteristics of the study cohort

Parameter	Study cohort (total *N* = 196)	
	*N*	%
Age		
< 55 years	144	73.5
55–64 years	44	22.4
> 64 years	8	4.1
HR status		
HR− (TNBC)	32	16.3
HR+	164	83.7
Clinical tumor stage		
cT1	1	0.5
cT2	111	56.6
cT3/cT4	84	42.9
Clinical lymph node stage		
cN−	94	48.0
cN+	102	52.0
Tumor type		
Ductal	135	68.9
Lobular	29	14.8
Other	18	9.2
Unknown	14	7.1
Menopausal status		
Pre	106	54.1
Peri	9	4.6
Post	79	40.3
Unknown	2	1.0
Allocated treatment		
Chemotherapy + ZA	95	48.5
Chemotherapy only	101	51.5

*HR* hormone receptor, *TNBC* triple-negative breast cancer, ZA zoledronic acid

### Pre-treatment immune markers and associations with pCR

**Fig. 2:**
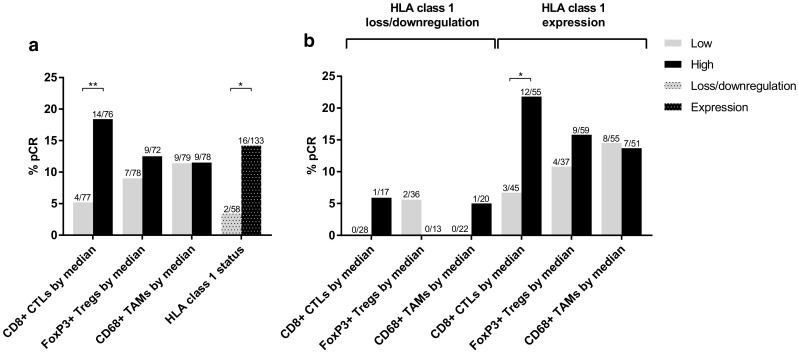
Response (pCR) to NAC. **a** Stratified on immune markers **b** Based on immune markers, stratified on HLA class 1 status. Loss or downregulation of HLA class 1 is defined as < 5% of tumor cells staining positive for HCA2 and HC10 (loss), or either HCA2 or HC10 (downregulation). Expression of HLA class 1 is defined as ≥ 5% of tumor cells staining positive for HCA2 and HC10. *Chi-square/Fisher’s exact test *p* < 0.05, **Chi-square test *p* ≤ 0.01

Successful staining and scoring of CD8+ T-cells, FoxP3+ T-cells and CD68+ macrophages was obtained in 82% (*n* = 160), 80% (*n* = 156), and 83% (*n* = 163) of biopsies, respectively. An overview of general statistics for each immune marker for the whole group and stratified on allocated treatment can be found in Online Resource 2. When the group of patients was divided based on the median cell count per mm^2^ in the tumor area, higher pCR rates were observed in patients with high (above median) CD8+ infiltrate compared to low (below median) CD8+ infiltrate (pCR: 18.4% in high CD8+ infiltrate vs. 5.2% in low CD8+ infiltrate; *p* = 0.01). No statistical differences were observed in pCR rates when patients were divided based on the number of FoxP3+ (pCR: 12.5% in high FoxP3+ infiltrate vs. 9.0% in low FoxP3+ infiltrate; *p* = 0.49) or CD68+ immune cells (pCR: 11.5% in high CD68+ infiltrate vs. 11.4% in low CD68+ infiltrate; *p* = 0.98) (Fig. 2 and Online Resource 3).

Successful staining and scoring of HLA class 1 was achieved in 91% (*n* = 178) and 87% (*n* = 170) of biopsies, respectively. Based on these data, the HLA class 1 status of the tumor was determined in 91% (*n* = 178). Tumors with loss or downregulation of HLA class 1 expression responded significantly worse to NAC than tumors with membranous HLA class 1 expression (pCR: 14.2% in tumors with HLA class 1 expression vs. 3.4% in tumors with HLA class 1 loss or downregulation; *p* = 0.03) (Fig. 2 and Online Resource 3).

The expression of HLA class 1 is a prerequisite for CD8+ CTLs to recognize tumor cells [36] and therefore the combination of these immune markers was evaluated further. In the presence of HLA class 1 expression, high CD8+ infiltrate was prognostic for pCR (pCR: 21.8% in high CD8+ infiltrate vs. 6.7% in low CD8+ infiltrate; *p* = 0.04), whereas when HLA class 1 expression was lost or downregulated, there was no benefit of high CD8+ infiltrate (pCR: 5.9% in high CD8+ infiltrate vs. 0% in low CD8+ infiltrate; *p* = 0.38). No such association was found for FoxP3+ (in presence of HLA class 1 expression: *p* = 0.54, loss or downregulation of HLA class 1 expression: *p* = 1.00) or CD68+ infiltrate (in presence of HLA class 1 expression: *p* = 0.90, loss or downregulation of HLA class 1 expression: *p* = 0.48) (Fig. 2 and Online Resource 3). The balance between immune effector cells and immune suppressive cells is often used as a prognostic and predictive measure [22, 37]. When the group of patients was divided into four categories based on the combination of CD8+ and FoxP3+ infiltrate status (i.e., CD8 low/FoxP3 low, CD8 high/FoxP3 low, CD8 low/FoxP3 high, CD8 high/FoxP3 high), no advantage for one group over the other was found (*p* = 0.14), neither when stratified on HLA class 1 status (in the presence of HLA class 1 expression: *p* = 0.43, loss or downregulation of HLA class 1 expression: *p* = 0.59).

The group of patients was then divided based on hormone receptor (HR) status, defined as negative when HER2, ER, and PR were negative (HR−/triple-negative breast cancer (TNBC)), and positive when ER and/or PR were positive (HR+). Patients with TNBC achieved more often a pCR (pCR: 26.7% (8 out of 30) in TNBC group vs. 8.2% (13 out of 159) in HR+ group; *p* = 0.008). Furthermore, the TNBC group more often had HLA class I expression (86.2% in TNBC group vs. 61.7% in HR+ group; *p* = 0.01) (Online Resource 1) and all seven patients with TNBC that achieved a pCR showed normal HLA class I expression, while six of these seven patients had strong CD8+ infiltrate. There were no differences in CD8+ or CD68+ infiltrate between the TNBC and HR+ group (*p* = 0.39 and *p* = 0.80, respectively), albeit that the numbers of FoxP3+ infiltrate were increased in the TNBC group (80.8% FoxP3+ high infiltrate in TNBC group vs. 43.8% FoxP3+ high infiltrate in HR+ group; *p* = 0.001) (Online Resource 1).

Allocated treatment-based analysis (chemotherapy plus or minus ZA), stratified on immune marker status, did not reveal any significant differences (Table 2) and neither did immune marker-based analysis, stratified on menopausal status (Online Resource 4). Immune marker-based analysis, stratified on HR status, showed that high CD8+ infiltrate and HLA expression are predictive for pCR in the HR+ group, but this is only a trend (pCR: 12.9% in high CD8+ infiltrate vs. 4.5% in low CD8+ infiltrate; *p* = 0.09 and pCR: 10.0% in tumors with HLA class 1 expression vs. 3.7% in tumors with HLA class 1 loss or downregulation; *p* = 0.21, respectively) (Online Resource 4).

**Table 2:** Allocated treatment-based analysis stratified on immune marker status

CD8+ CTLs									FoxP3+ Tregs							
	Low (< median)				High (> median)				Low (< median)				High (> median)			
	No pCR		pCR		No pCR		pCR		No pCR		pCR		No pCR		pCR	
	N	%	N	%	N	%	N	%	N	%	N	%	N	%	N	%
Allocated treatment																
ZA+	30	96.8%	1	3.2%	37	84.1%	7	15.9%	37	92.5%	3	7.5%	31	83.8%	6	16.2%
ZA−	43	93.5%	3	6.5%	25	78.1%	7	21.9%	34	89.5%	4	10.5%	32	91.4%	3	8.6%
Total	77				76				78				72			
*p* value	0.64				0.51				0.71				0.48			

	CD68+ TAMs								HLA class 1 status							
	Low (< median)				High (> median)				Loss + downregulation				Expression			
	No pCR		pCR		No pCR		pCR		No pCR		pCR		No pCR		pCR	
	*N*	%	*N*	%	*N*	%	*N*	%	*N*	%	*N*	%	*N*	%	*N*	%
Allocated treatment																
ZA+	31	91.2%	3	8.8%	36	85.7%	6	14.3%	26	92.9%	2	7.1%	48	87.3%	7	12.7%
ZA−	39	86.7%	6	13.3%	33	91.7%	3	8.3%	30	100.0%	0	0.0%	49	84.5%	9	15.5%
Total	79				78				58				113			
*p* value	0.73				0.49				0.23				0.67			

Loss or downregulation of HLA class 1 is defined as < 5% of tumor cells staining positive for HCA2 and HC10 (loss), or either HCA2 or HC10 (downregulation). Expression of HLA class 1 is defined as ≥ 5% of tumor cells staining positive for HCA2 and HC10. *p* values represent Chi square of Fisher’s exact tests. *CTLs* cytotoxic T-cells, *TAMs* tumor-associated macrophages, *Tregs* regulatory T-cells, *ZA* zoledronic acid

Finally, multivariable analyses were performed to analyze the effect of each separate immune marker when corrected for hormonal status (positive or negative). This showed that the effect of CD8+ infiltrate on pCR was independent of hormonal status (OR 3.95, 95% CI 1.21–12.90, *p* = 0.02). Also, HLA class 1 expression influenced pCR rate irrespective of hormonal status (OR 3.78, 95% CI 0.82–17.42, *p* = 0.09), albeit this was a trend. No effect of FoxP3+ and CD68+ infiltrate was found (OR 0.89, 95% CI 0.28–2.85, *p* = 0.85 and OR 0.97, 95% CI 0.35–2.73, *p* = 0.96, respectively).

### Pre-treatment immune markers and associations with DFS

**Fig. 3:**
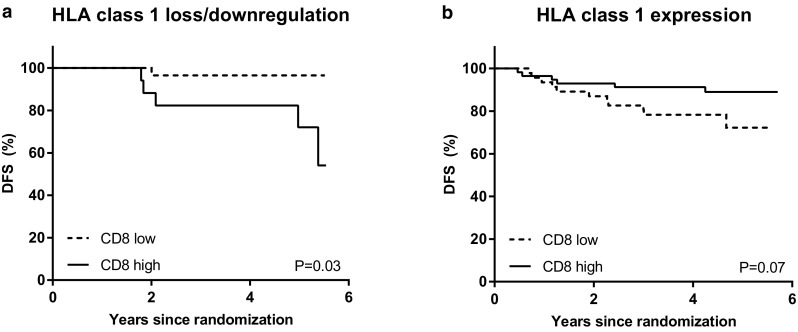
Kaplan–Meier survival estimates based on CD8+ TILs, stratified on HLA class 1 status. **a** Effect of CD8+ tumor infiltrate on DFS in patients with HLA class 1 loss or downregulation. **b** Effect of CD8+ tumor infiltrate on DFS in patients with HLA class 1 expression. *p* values represent log-rank survival test

**Fig. 4:**
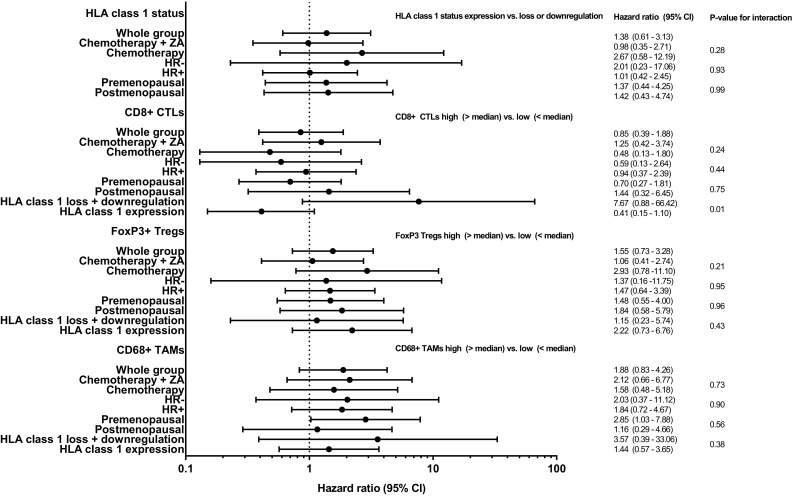
Forest plot with immune marker-based (subgroup) analyses of DFS

Median follow-up was 4.6 years (cut-off date December 1, 2016). Upon stratification based on the median cell count per mm^2^ in the tumor area, a numerical but not significant trend towards a lower rate of DFS was observed in patients with high CD68+ infiltrate (hazard ratio 1.88, 95% CI 0.83–4.26, *p* = 0.13). No statistical differences in DFS between patients with high or low CD8+ T-cell tumor infiltrate (hazard ratio 0.85, 95% CI 0.39–1.88, *p* = 0.70), high or low FoxP3+ T-cell tumor infiltrate (hazard ratio 1.55, 95% CI 0.73–3.28, *p* = 0.25), or expression or loss/downregulation of HLA class 1 of tumor cells were found (hazard ratio 1.38, 95% CI 0.61–3.13, *p* = 0.44). However, in the presence of HLA class 1 expression, patients with a tumor that was strongly infiltrated with CD8+ T-cells displayed a survival benefit, whereas in the absence or downregulation of HLA class 1 expression, this effect was lost (p for interaction 0.01; hazard ratio 0.41, 95% CI 0.15–1.10, *p* = 0.08 and hazard ratio 7.67, 95% CI 0.88–66.4, *p* = 0.07, respectively) (Figs. 3, 4).

**Fig. 5:**
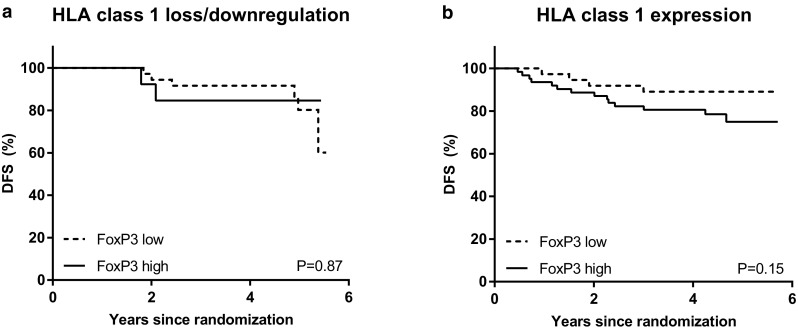
Kaplan–Meier survival estimates based on FoxP3+ TILs, stratified on HLA class 1 status. **a** Effect of FoxP3+ tumor infiltrate on DFS in patients with HLA class 1 loss or downregulation. **b** Effect of FoxP3+ tumor infiltrate on DFS in patients with HLA class 1 expression. *p* values represent log-rank survival test

Similar analyses were performed for CD68+ and FoxP3+ infiltrate. CD68+ infiltrate was shown not to be prognostic in either subgroup (Fig. 4) and although FoxP3+ infiltrate was neither prognostic in patients with loss or downregulation of HLA class 1 expression, it tended to be inversely prognostic for DFS in patients with HLA class 1 expression (hazard ratio 2.22, 95% CI 0.73–6.76, *p* = 0.16), fitting with its immune suppressive role (Figs. 4, 5).

**Fig. 6:**
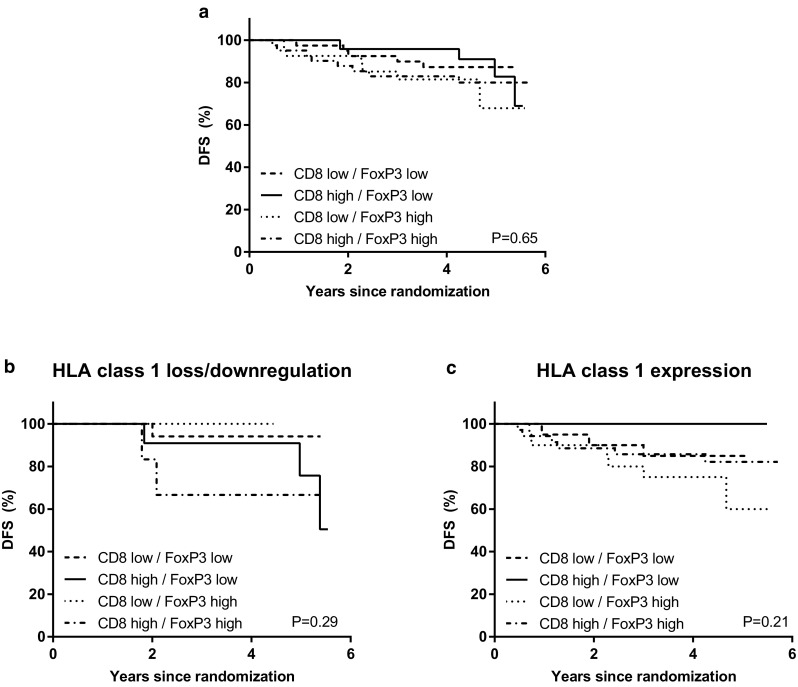
Kaplan–Meier survival estimates based on the combination of CD8+ infiltrate and FoxP3+ infiltrate status. **a** Effect of the combination on DFS in the whole group. **b** Effect of the combination on DFS in patients with HLA class 1 loss or downregulation. **c** Effect of the combination on DFS in patients with HLA class 1 expression. *p* values represent log-rank survival test

When the group of patients was then divided into four categories based on the combination of CD8+ infiltrate and FoxP3+ infiltrate status as described above, no prognostic effect could be observed in the whole group (log-rank *p* = 0.65). When stratified on HLA class 1 status, CD8+ high/FoxP3+ low infiltrate resulted in an improved DFS in the HLA class 1 expression group, although not significantly (log-rank *p* = 0.21, p for interaction 0.69) (Fig. 6).

The ratio CD8+/CD68+ infiltrating immune cells was not prognostic in the whole group (hazard ratio 0.66, 95% CI 0.29–1.53, *p* = 0.33), neither when stratified on HLA class 1 status (p for interaction 0.86).

Additional subgroup analysis stratified on allocated treatment, HR status, and menopausal status did not reveal any significant differences except an inversely prognostic effect of CD68+ TAMs on DFS in premenopausal women (hazard ratio 2.85, CI 1.03–7.88, *p* = 0.04). However, the interaction test was not significant (*p* = 0.56) (Fig. 4). In HR+ patients, there was a trend for a better DFS (*p* = 0.19); however, HLA class 1 expression in combination with high CD8+ infiltrate was associated with an improved DFS, irrespective of the HR status (Online Resource 5).

## Discussion

Our study shows that CD8+ TIL infiltrate and HLA class 1 status as individual markers are associated with higher pCR rates after NAC. Neither of the immune markers was prognostic for DFS. However, when CD8+ TIL infiltrate was combined with HLA class 1 status, it became clear that higher pCR rates and a better DFS were detected in patients with a strongly CD8+ T-cell infiltrated tumor which had retained a strong expression of HLA class 1. To our knowledge, this is the first study investigating and defining the role of HLA class 1 expression in pCR after NAC.

Previous studies have concordantly proven TILs to be prognostic for outcome after NAC. However, the prognostic value of the different TIL subtypes is less clear. We found that high intratumoral CD8+ infiltrate, but not CD68+ and FoxP3+ infiltrate, correlates with a higher pCR. Furthermore, we observed a better pathological response of tumors strongly expressing HLA class 1. We did not observe a direct relation between HLA class 1 status and survival as de Kruijf et al. did for chemotherapy treated patients. However, this might be explained by the fact that they studied a slightly different patient population (e.g., also patients with HER2-positive tumors) and when chemotherapy was utilized, this was done in the adjuvant setting. In the NAC group, a beneficial effect on pCR and DFS was only observed when a strong infiltration of CD8+ T-cells coincided with a strong expression of HLA class 1. This is explained by the fact that CD8+ CTLs are only capable of recognizing malignant cells if they express HLA class 1. Consequently, downregulation of HLA class 1 is commonly found in tumors as an immune escape mechanism [38].

TNBC is known to correlate with the presence of immune cells and a better response to chemotherapy than ER-positive breast cancer [7]. Indeed, the patient group with TNBC had a higher chance of achieving a pCR than those without TNBC. The small size of this group precludes strong statistically significant differences; however, the TNBC group more often had normal HLA class I expression and all TNBC patients achieving a pCR showed normal HLA class I expression, while all except one had stronger CD8+ T-cell infiltrate. There were no differences in CD8+ or CD68+ infiltration between patients with TNBC or without, albeit that the numbers of FoxP3+ cells were increased in TNBC. The latter, however, cannot explain why patients with TNBC would have relatively more pCRs. In the multivariable analyses with correction for hormonal status, the effect of CD8+ infiltrate on pCR was still significant and the effect of HLA class 1 status borderline significant.

Literature suggests that ZA is capable of eliciting an antitumor effect by modulating the immune system, specifically on gamma-delta T-cells [28] and on the recruitment and infiltration of TAMs [39–41]. Although we did not look into post-treatment immune changes in the tumor specimen, analysis of pre-treatment immune marker status did not reveal a relation between the number of TAMs and benefit from neoadjuvant ZA added to standard chemotherapy. Also, no treatment benefit of ZA for the other immune markers on pCR or DFS was found. Potentially, these pre-clinical data do not easily translate into the human setting. Another option is that because ZA as an adjunct to NAC did not improve pathological or clinical response to chemotherapy [27], no predictive immune biomarkers were found either. In addition, we did not study the effect of gamma-delta T-cells in the tumors.

Our study investigated TILs and TAMs that were located in the tumor cell nests. An international TIL-working group recently postulated recommendations for evaluation of TILs on H&E-stained slides [8–10]. As intra- and peritumoral lymphocytes are hard to observe on H&E slides, the recommendations are based on TIL evaluation in the stromal compartment adjacent to the tumor in biopsies. However, with regard to the interaction between TILs and tumor cells, there is no biological rationale to use stromal TILs when intratumoral TILs can be visualized with immunohistochemistry [9, 10]. For this reason, in our study, intratumoral TILs and TAMs were assessed.

Our study was limited regarding sample size as the study cohort was not prospectively powered for detecting statistical differences in pCR or DFS based on immune markers. Our current findings should be validated in an independent cohort with a similar population and treatment setting. Our current study was too small in sample size to do an adequate analysis of the most appropriate cut-off value. For this reason, the median of TIL and TAM cell counts was used for CD8+/FoxP3+ and CD68+ status, respectively, similar to others [42, 43]. One of the goals of a validation study should be to further investigate what an optimal cut-off value of cell counts may be for intratumoral CD8+ lymphocytes and the level of HLA class I downregulation that is still acceptable.

## Conclusions

In summary, the new important finding for the clinic is that a strong baseline infiltration of early breast tumors with CD8+ T-cells is related to a higher pCR rate and a better DFS after NAC when the tumor cells have retained expression of HLA class 1. Ultimately, it shows that the chemotherapy effect is also dependent on the immune system.

## Electronic supplementary material

Below is the link to the electronic supplementary material.

ESM_1 (Online Resource 1): Patient and tumor characteristics of successfully scored patients for each separate immune marker (table). Supplementary material 1 (PDF 443 KB)

ESM_2 (Online Resource 2): Overview of general statistics for each immune marker (whole group and stratified on allocated treatment) (table). Supplementary material 2 (PDF 332 KB)

ESM_3 (Online Resource 3): Response (pCR) to chemotherapy a Stratified on immune markers and b Based on immune markers, stratified on HLA class 1 status (table). Supplementary material 3 (PDF 421 KB)

ESM_4 (Online Resource 4): Immune marker-based analyses stratified on HR status and menopausal status (table). Supplementary material 4 (PDF 418 KB)

ESM_5 (Online Resource 5): Kaplan–Meier survival estimates based on HR status and based on CD8+ tumor infiltrate, stratified on HLA class 1 status (figure). Supplementary material 5 (PDF 314 KB)

## Data Availability

The datasets used and/or analyzed during the current study are available from the corresponding author on reasonable request.

## Abbreviations

CTLs: Cytotoxic T-cells
DAB: 3,3′-Diaminobenzidine
DFS: Disease-free survival
ER: Estrogen receptor
FFPE: Formalin-fixed paraffin-embedded
H&E: Hematoxylin and eosin stained
HR: Hormone receptor
NAC: Neoadjuvant chemotherapy
pCR: Pathological complete response
PR: Progesterone receptor
TAMs: Tumor-associated macrophages
TILs: Tumor-infiltrating lymphocytes
TNBC: Triple-negative breast cancer
Tregs: Regulatory T-cells
ZA: Zoledronic acid
