# Supplementary material for: Strong CD8+ lymphocyte infiltration in combination with expression of HLA class I is associated with better tumor control in breast cancer patients treated with neoadjuvant chemotherapy
Source: Breast Cancer Res Treat. 2019 Mar 13;175(3):605–15. doi: 10.1007/s10549-019-05195-y (PMC6534526; doi:10.1007/s10549-019-05195-y)
Supplement: Supplementary file 1 — ESM_1 (Online Resource 1): Patient and tumor characteristics of successfully scored patients for each separate immune marker (table). Supplementary material 1 (PDF 443 KB) [file 10549_2019_5195_MOESM1_ESM.pdf]

Online Resource 1

Strong CD8+ lymphocyte infiltration in combination with expression of HLA class I is associated with better tumor control in breast cancer patients treated with neoadjuvant chemotherapy

Journal: Breast Cancer Research and Treatment

Authors: A.F. de Groot [1], E.J. Blok[1,2], A. Charehbili[1,2], C.C. Engels[2], V.T.H.B.M. Smit[3], N.G. Dekker-Ensink[2], H. Putter[4], E. Meershoek - Klein Kranenbarg[2], C.J.H. van de Velde[2], G.J. Liefers[2], J.W.R. Nortier[1], P.J.K. Kuppen[2], S.H. van der Burg[1], J.R. Kroep[1]

Departments of Medical Oncology[1], Surgery[2], Pathology[3], Statistics[4], Leiden University Medical Center, Leiden, The Netherlands

Corresponding author: Judith R. Kroep, M.D., Ph.D. (j.r.kroep@lumc.nl)

Online Resource 1 Patient and tumor characteristics of successfully scored patients for each separate immune marker

Parameter		CD8+ CTLs by median (n=160)				FoxP3+ Tregs by median (n=156)				CD68+ TAMs by median (n=163)				HLA class 1 status (n=178)			
		Low (< median)		High (> median)		Low (< median)		High (> median)		Low (< median)		High (> median)		Loss or downregulation		Expression	
		N	%	N	%	N	%	N	%	N	%	N	%	N	%	N	%
Age	<55 years	59	50.0%	59	50.0%	59	51.8%	55	48.2%	64	52.9%	57	47.1%	**38**	**29.0%**	**93**	**71.0%**
	55-64 years	17	50.0%	17	50.0%	16	47.1%	18	52.9%	15	42.9%	20	57.1%	**19**	**48.7%**	**20**	**51.3%**
	>64 years	4	50.0%	4	50.0%	3	37.5%	5	62.5%	3	42.9%	4	57.1%	**4**	**50.0%**	**4**	**50.0%**
HR status	HR- (TNBC)	11	42.3%	15	57.7%	**5**	**19.2%**	**21**	**80.8%**	12	48.0%	13	52.0%	**4**	**13.8%**	**25**	**86.2%**
	HR+	69	51.5%	65	48.5%	**73**	**56.2%**	**57**	**43.8%**	70	50.7%	68	49.3%	**57**	**38.3%**	**92**	**61.7%**
Clinical tumor stage	cT1/ cT2	43	44.8%	53	55.2%	37	43.0%	49	57.0%	47	48.5%	50	51.5%	**26**	**25.2%**	**77**	**74.8%**
	cT3/cT4	37	57.8%	27	42.2%	41	58.6%	29	41.4%	35	53.0%	31	47.0%	**35**	**46.7%**	**40**	**53.3%**
Clinical lymph node stage	cN-	42	56.8%	32	43.2%	**43**	**60.6%**	**28**	**39.4%**	43	58.1%	31	41.9%	**36**	**42.9%**	**48**	**57.1%**
	cN+	38	44.2%	48	55.8%	**35**	**41.2%**	**50**	**58.8%**	39	43.8%	50	56.2%	**25**	**26.6%**	**69**	**73.4%**
Tumor type	Ductal	52	46.4%	60	53.6%	**45**	**41.7%**	**63**	**58.3%**	61	53.5%	53	46.5%	35	28.2%	89	71.8%
	Lobular	9	40.9%	13	59.1%	**19**	**73.1%**	**7**	**26.9%**	9	36.0%	16	64.0%	12	44.4%	15	55.6%
	Other	10	66.7%	5	33.3%	**7**	**70.0%**	**3**	**30.0%**	7	53.8%	6	46.2%	7	50.0%	7	50.0%
Menopausal status	Pre	45	49.5%	46	50.5%	41	48.8%	43	51.2%	**51**	**57.3%**	**38**	**42.7%**	31	32.0%	66	68.0%
	Peri	1	20.0%	4	80.0%	2	33.3%	4	66.7%	**5**	**83.3%**	**1**	**16.7%**	2	28.6%	5	71.4%
	Post	33	53.2%	29	46.8%	34	53.1%	30	46.9%	**26**	**39.4%**	**40**	**60.6%**	28	38.9%	44	61.1%
Allocated treatment	Chemotherapy + ZA	33	42.3%	45	57.7%	40	50.6%	39	49.4%	36	46.2%	42	53.8%	30	34.9%	56	65.1%
	Chemotherapy only	47	57.3%	35	42.7%	38	49.4%	39	50.6%	46	54.1%	39	45.9%	31	33.7%	61	66.3%

Loss or downregulation of HLA class 1 is defined as <5% of tumor cells staining positive for HCA2 and HC10 (loss), or either HCA2 or HC10 (downregulation). Expression of HLA class 1 is defined as ≥5% of tumor cells staining positive for HCA2 and HC10. **Bold/underline**: Chi-square or Fisher's exact p-value <0.05. *CTLs* cytotoxic T-cells, *HR* hormone receptor, *TAMs* tumor-associated macrophages, *TNBC* triple negative breast cancer, *Tregs* regulatory T-cells, *ZA* zoledronic acid
