# Supplementary material for: Strong CD8+ lymphocyte infiltration in combination with expression of HLA class I is associated with better tumor control in breast cancer patients treated with neoadjuvant chemotherapy
Source: Breast Cancer Res Treat. 2019 Mar 13;175(3):605–15. doi: 10.1007/s10549-019-05195-y (PMC6534526; doi:10.1007/s10549-019-05195-y)
Supplement: Supplementary file 2 — ESM_2 (Online Resource 2): Overview of general statistics for each immune marker (whole group and stratified on allocated treatment) (table). Supplementary material 2 (PDF 332 KB) [file 10549_2019_5195_MOESM2_ESM.pdf]

Online Resource 2

Strong CD8+ lymphocyte infiltration in combination with expression of HLA class I is associated with better tumor control in breast cancer patients treated with neoadjuvant chemotherapy

Journal: Breast Cancer Research and Treatment

Authors: A.F. de Groot [1], E.J. Blok[1,2], A. Charehbili[1,2], C.C. Engels[2], V.T.H.B.M. Smit[3], N.G. Dekker-Ensink[2], H. Putter[4], E. Meershoek - Klein Kranenbarg[2], C.J.H. van de Velde[2], G.J. Liefers[2], J.W.R. Nortier[1], P.J.K. Kuppen[2], S.H. van der Burg[1], J.R. Kroep[1]

Departments of Medical Oncology[1], Surgery[2], Pathology[3], Statistics[4], Leiden University Medical Center, Leiden, The Netherlands

Corresponding author: Judith R. Kroep, M.D., Ph.D. (j.r.kroep@lumc.nl)

Online Resource 2 Overview of general statistics for each immune marker (whole group and stratified on allocated treatment)

			CD8+ CTLs (per mm^2)	FoxP3+ Tregs (per mm^2)	CD68+ TAMs (per mm^2)
**Whole group**		Number (successful staining and scoring)	160	156	163
		Mean	536.8	87.5	592.3
		Median	269.0	29.4	352.2
		Standard deviation	853.3	134.1	733.7
		Minimum	0.00	0.00	2.00
		Maximum	5883.4	681.3	3858.2
**Allocated treatment**	**Chemotherapy + ZA**	Number (successful staining and scoring)	78	79	78
		Mean	466.3	84.8	548.6
		Median	291.4	25.2	382.3
		Standard deviation	557.1	125.0	615.9
		Minimum	0.70	0.00	10.6
		Maximum	3464.6	632.8	3208.0
	**Chemotherapy only**	Number (successful staining and scoring)	82	77	85
		Mean	603.9	90.2	632.5
		Median	184.5	32.7	270.4
		Standard deviation	1060.6	143.6	828.8
		Minimum	0.00	0.00	2.00
		Maximum	5883.4	681.3	3858.2

Scores represent number of immune cells per mm^2 tumor area. *CTLs* cytotoxic T-cells, *TAMs* tumor-associated macrophages, *Tregs* regulatory T-cells, *ZA* zoledronic acid
