# Supplementary material for: Strong CD8+ lymphocyte infiltration in combination with expression of HLA class I is associated with better tumor control in breast cancer patients treated with neoadjuvant chemotherapy
Source: Breast Cancer Res Treat. 2019 Mar 13;175(3):605–15. doi: 10.1007/s10549-019-05195-y (PMC6534526; doi:10.1007/s10549-019-05195-y)
Supplement: Supplementary file 3 — ESM_3 (Online Resource 3): Response (pCR) to chemotherapy a Stratified on immune markers and b Based on immune markers, stratified on HLA class 1 status (table). Supplementary material 3 (PDF 421 KB) [file 10549_2019_5195_MOESM3_ESM.pdf]

Online Resource 3

Strong CD8+ lymphocyte infiltration in combination with expression of HLA class I is associated with better tumor control in breast cancer patients treated with neoadjuvant chemotherapy

Journal: Breast Cancer Research and Treatment

Authors: A.F. de Groot [1], E.J. Blok[1,2], A. Charehbili[1,2], C.C. Engels[2], V.T.H.B.M. Smit[3], N.G. Dekker-Ensink[2], H. Putter[4], E. Meershoek - Klein Kranenbarg[2], C.J.H. van de Velde[2], G.J. Liefers[2], J.W.R. Nortier[1], P.J.K. Kuppen[2], S.H. van der Burg[1], J.R. Kroep[1]

Departments of Medical Oncology[1], Surgery[2], Pathology[3], Statistics[4], Leiden University Medical Center, Leiden, The Netherlands

Corresponding author: Judith R. Kroep, M.D., Ph.D. (j.r.kroep@lumc.nl)

Online Resource 3 Response (pCR) to chemotherapy **a** Stratified on immune markers and **b** Based on immune markers, stratified on HLA class 1 status

a

		No pCR		pCR		Total	P-value
		**N**	**%**	**N**	**%**	**N**	
**CD8+ CTLs by median**	Low (< median)	73	94.8%	4	5.2%	153	0.01
	High (> median)	62	81.6%	14	18.4%		
**FoxP3+ Tregs by median**	Low (< median)	71	91.0%	7	9.0%	150	0.49
	High (> median)	63	87.5%	9	12.5%		
**CD68+ TAMs by median**	Low (< median)	70	88.6%	9	11.4%	157	0.98
	High (> median)	69	88.5%	9	11.5%		
**HLA class 1 status**	Loss or downregulation	56	96.6%	2	3.4%	171	0.03
	Expression	97	85.8%	16	14.2%		

b

		HLA class 1 status												
		Loss or downregulation						Expression						
		No pCR		pCR		Total	P-value	No pCR		pCR		Total	P-value
		N	%	N	%	N		N	%	N	%	N	
**CD8+ CTLs by median**	Low (< median)	28	100.0%	0	0.0%	45	0.38	42	93.3%	3	6.7%	100	0.04
	High (> median)	16	94.1%	1	5.9%			43	78.2%	12	21.8%		
**FoxP3+ Tregs by median**	Low (< median)	34	94.4%	2	5.6%	49	1.00	33	89.2%	4	10.8%	96	0.54
	High (> median)	13	100.0%	0	0.0%			50	84.7%	9	15.3%		
**CD68+ TAMs by median**	Low (< median)	22	100.0%	0	0.0%	42	0.48	47	85.5%	8	14.5%	106	0.90
	High (> median)	19	95.0%	1	5.0%			44	86.3%	7	13.7%		

Loss or downregulation of HLA class 1 is defined as <5% of tumor cells staining positive for HCA2 and HC10 (loss), or either HCA2 or HC10 (downregulation). Expression of HLA class 1 is defined as ≥5% of tumor cells staining positive for HCA2 and HC10. P-values represent Chi-square or Fisher's exact tests. *CTLs* cytotoxic T-cells, *pCR* pathological complete response, *TAMs* tumor-associated macrophages, *Tregs* regulatory T-cells
