# Supplementary material for: Strong CD8+ lymphocyte infiltration in combination with expression of HLA class I is associated with better tumor control in breast cancer patients treated with neoadjuvant chemotherapy
Source: Breast Cancer Res Treat. 2019 Mar 13;175(3):605–15. doi: 10.1007/s10549-019-05195-y (PMC6534526; doi:10.1007/s10549-019-05195-y)
Supplement: Supplementary file 4 — ESM_4 (Online Resource 4): Immune marker-based analyses stratified on HR status and menopausal status (table). Supplementary material 4 (PDF 418 KB) [file 10549_2019_5195_MOESM4_ESM.pdf]

Online Resource 4

Strong CD8+ lymphocyte infiltration in combination with expression of HLA class I is associated with better tumor control in breast cancer patients treated with neoadjuvant chemotherapy

Journal: Breast Cancer Research and Treatment

Authors: A.F. de Groot [1], E.J. Blok[1,2], A. Charehbili[1,2], C.C. Engels[2], V.T.H.B.M. Smit[3], N.G. Dekker-Ensink[2], H. Putter[4], E. Meershoek - Klein Kranenbarg[2], C.J.H. van de Velde[2], G.J. Liefers[2], J.W.R. Nortier[1], P.J.K. Kuppen[2], S.H. van der Burg[1], J.R. Kroep[1]

Departments of Medical Oncology[1], Surgery[2], Pathology[3], Statistics[4], Leiden University Medical Center, Leiden, The Netherlands

Corresponding author: Judith R. Kroep, M.D., Ph.D. (j.r.kroep@lumc.nl)

Online Resource 4 Immune marker-based analyses stratified on HR status and menopausal status

		CD8+ CTLs					FoxP3+ Tregs					CD68+ TAMs					HLA class 1 status				
		**Low (< median)**		**High (> median)**		P-value	**Low (< median)**		**High (> median)**		P-value	**Low (< median)**		**High (> median)**		P-value	**Loss + downregulation**		**Expression**		P-value
		pCR		pCR			pCR		pCR			pCR		pCR			pCR		pCR		
		N	%	N	%		N	%	N	%		N	%	N	%		N	%	N	%	
HR status	HR- (TNBC)	1	10.0%	6	42.9%	0.17	1	20.0%	6	31.6%	1.00	4	36.4%	4	33.3%	1.00	0	0.0%	7	30.4%	0.55
	HR+	3	4.5%	8	12.9%	0.09	6	8.2%	3	5.7%	0.73	5	7.4%	5	7.6%	1.00	2	3.7%	9	10.0%	0.21
Menopausal status	Pre	2	4.8%	7	15.9%	0.16	3	7.3%	3	7.7%	1.00	6	12.2%	2	5.6%	0.46	1	3.4%	7	11.1%	0.43
	Peri	0	0.0%	2	50.0%	1.00	1	50.0%	2	50.0%	1.00	1	20.0%	1	100.0%	0.33	1	50.0%	2	40.0%	1.00
	Post	1	3.0%	5	18.5%	0.08	2	5.9%	4	14.3%	0.40	2	8.0%	5	12.8%	0.70	0	0.0%	6	14.0%	0.08

Loss or downregulation of HLA class 1 is defined as <5% of tumor cells staining positive for HCA2 and HC10 (loss), or either HCA2 or HC10 (downregulation). Expression of HLA class 1 is defined as ≥5% of tumor cells staining positive for HCA2 and HC10. P-values represent Chi-square of Fisher's exact tests. *CTLs* cytotoxic T-cells, *HR* hormone receptor, *pCR* pathological complete response, *TAMs* tumor-associated macrophages, *TNBC* triple negative breast cancer, *Tregs* regulatory T-cells
