# Supplementary material for: Strong CD8+ lymphocyte infiltration in combination with expression of HLA class I is associated with better tumor control in breast cancer patients treated with neoadjuvant chemotherapy
Source: Breast Cancer Res Treat. 2019 Mar 13;175(3):605–15. doi: 10.1007/s10549-019-05195-y (PMC6534526; doi:10.1007/s10549-019-05195-y)
Supplement: Supplementary file 5 — ESM_5 (Online Resource 5): Kaplan–Meier survival estimates based on HR status and based on CD8+ tumor infiltrate, stratified on HLA class 1 status (figure). Supplementary material 5 (PDF 314 KB) [file 10549_2019_5195_MOESM5_ESM.pdf]

Online Resource 5

Strong CD8+ lymphocyte infiltration in combination with expression of HLA class I is associated with better tumor control in breast cancer patients treated with neoadjuvant chemotherapy

Journal: Breast Cancer Research and Treatment

Authors: A.F. de Groot [1], E.J. Blok[1,2], A. Charehbili[1,2], C.C. Engels[2], V.T.H.B.M. Smit[3], N.G. Dekker-Ensink[2], H. Putter[4], E. Meershoek - Klein Kranenbarg[2], C.J.H. van de Velde[2], G.J. Liefers[2], J.W.R. Nortier[1], P.J.K. Kuppen[2], S.H. van der Burg[1], J.R. Kroep[1]

Departments of Medical Oncology[1], Surgery[2], Pathology[3], Statistics[4], Leiden University Medical Center, Leiden, The Netherlands

Corresponding author: Judith R. Kroep, M.D., Ph.D. (j.r.kroep@lumc.nl)

Online Resource 5 Kaplan-Meier survival estimates based on HR status and based on CD8+ tumor infiltrate, stratified on HLA class 1 status

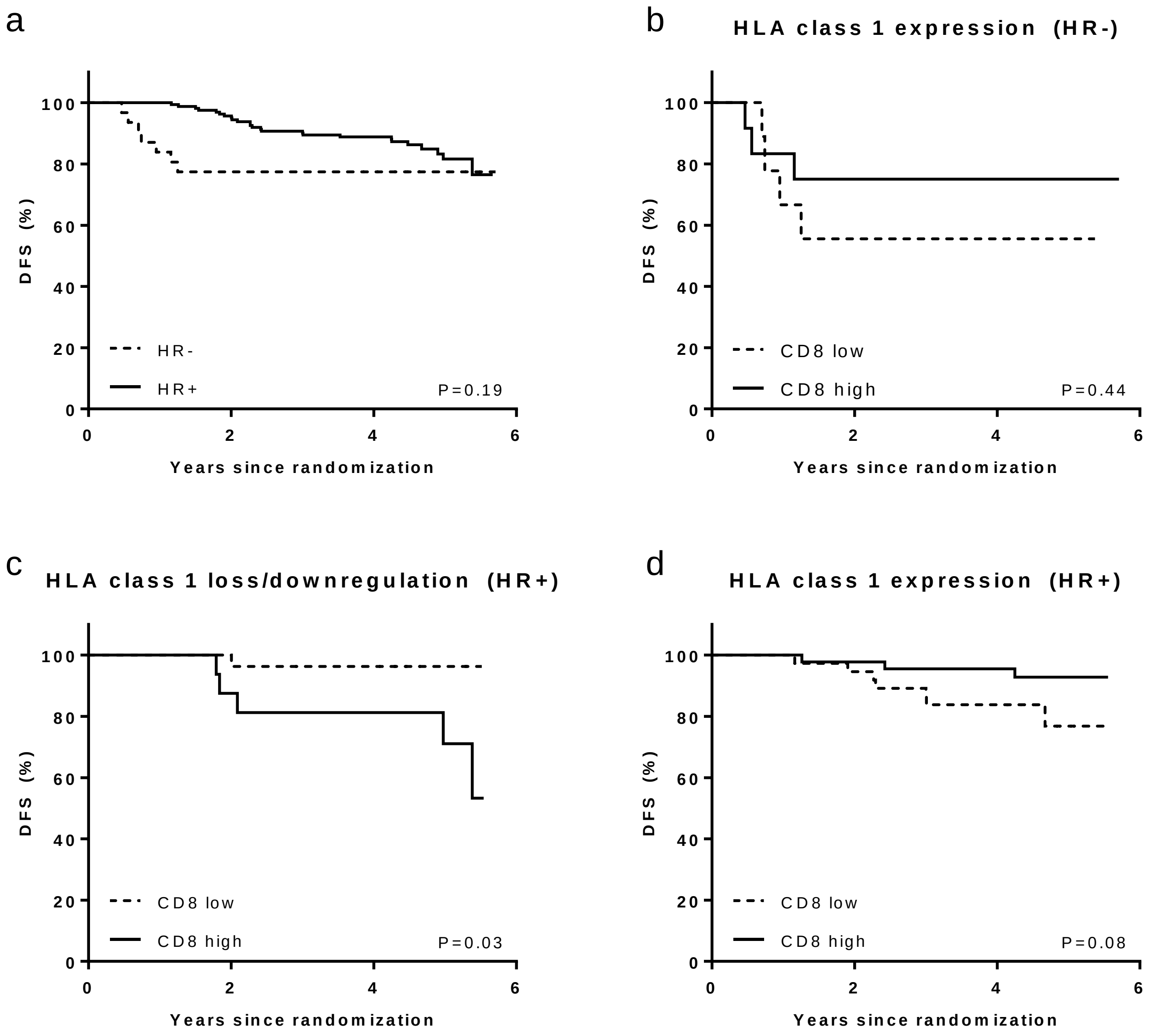

a Effect of HR status on DFS (hazard ratio 0.57, 95% CI 0.25-1.32, p=0.19) **b** Effect of CD8+ tumor infiltrate on DFS in patients with HR- disease with HLA class 1 expression (hazard ratio 0.56, 95% CI 0.12-2.49, p=0.44; Kaplan-Meier survival estimate and Cox regression analysis not possible for HLA class 1 loss or downregulation due to small numbers; p for interaction 0.99) **c** Effect of CD8+ tumor infiltrate on DFS in patients with HR+ disease with HLA class 1 loss or downregulation (hazard ratio 7.51, 95% CI 0.87-65.20, p=0.07) **d** Effect of CD8+ tumor infiltrate on DFS in patients with HR+ disease with HLA class 1 expression (hazard ratio 0.32, 95% CI 0.08-1.24, p=0.098). P for interaction 0.01. P-values in figure represent log-rank survival test
